# Disseminated Mycobacterial Infection as a Sequelae of Bladder Biopsy

**DOI:** 10.7759/cureus.18711

**Published:** 2021-10-12

**Authors:** Kitson Deane, Paula Rodriguez, Dhiviyan Valentine, Donald Hathaway, Fares Hosseinzadeh

**Affiliations:** 1 Medicine, Woodhull Medical Center, Brooklyn, USA; 2 Emergency Medicine, Lincoln Medical and Mental Health Center, Bronx, USA; 3 Medicine, St George's University, St George's, GRD

**Keywords:** hepatitis, mycobacterium bovis, bcg vaccine, mycobacterium, immunocompetent

## Abstract

Disseminated *Mycobacterium *infections have been commonly documented in the immunocompromised and patients who undergo treatment for non-muscle invasive bladder cancer with the Bacillus Calmette-Guerin vaccine; however, it was unique to our patient’s case that she had no record of receiving vaccination in her native country, was immunocompetent, and had exposure to bovine livestock before immigrating to the United States.

A 57-year-old woman with no significant medical history presented with complaints of abdominal pains and yellowing of her skin. Laboratory workup was consistent with cholestatic hepatitis. One month prior to presentation, she underwent biopsy and culture of an unspecified bladder mass, which turned out to be positive for *Mycobacterium bovis*. All antituberculosis medications were discontinued, without improvement of her symptoms and hepatic function tests. Subsequent liver biopsy showed the presence of granulomas with acid-fast bacilli; hence, disseminated infection was highly suspected. Multiple sputum cultures and quantiferon tests were negative, and other diagnostic tests were unremarkable. Initiation of appropriate antibiotics resulted in marked symptomatic improvement and gradual normalization of hepatic function parameters.

Disseminated mycobacterial infection may present differently in patients; however, it is important to note that the source of primary infection may vary. Prompt diagnosis and treatment of these pathogens may lead to improved outcomes.

## Introduction

The *Mycobacterium* genus consists of highly virulent pathogens known for causing a wide range of diseases including tuberculosis and leprosy. In some cases, mycobacterium can cause disseminated infections, which lead to devastating consequences such as multiorgan involvement and death. Disseminated infection from the bladder has been well described due to intravesical instillation during the treatment of non-muscle invasive bladder cancer with Bacillus Calmette-Guerin (BCG) vaccines formulated with *Mycobacterium bovis* (MB) and less commonly in patients with immunodeficiencies who receive the BCG vaccine for tuberculosis prevention [1]. We report a peculiar case of disseminated mycobacterium infection with involvement of the liver in an immunocompetent patient with no record of ever having received the BCG vaccine for prophylactic or therapeutic purposes.

## Case presentation

**Figure 1:**
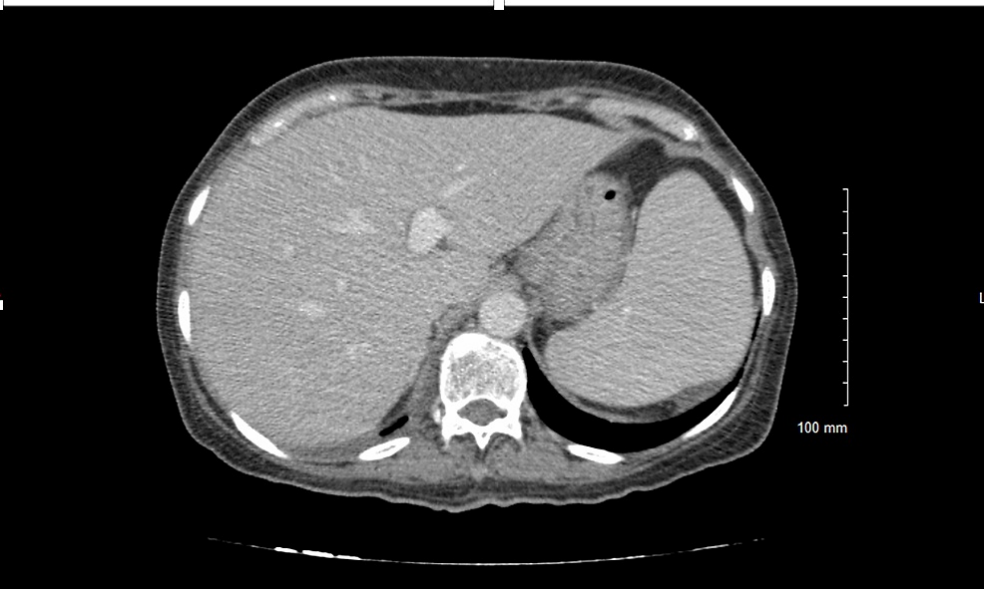
CT scan of the abdomen showing hepatosplenomegaly

**Figure 2:**
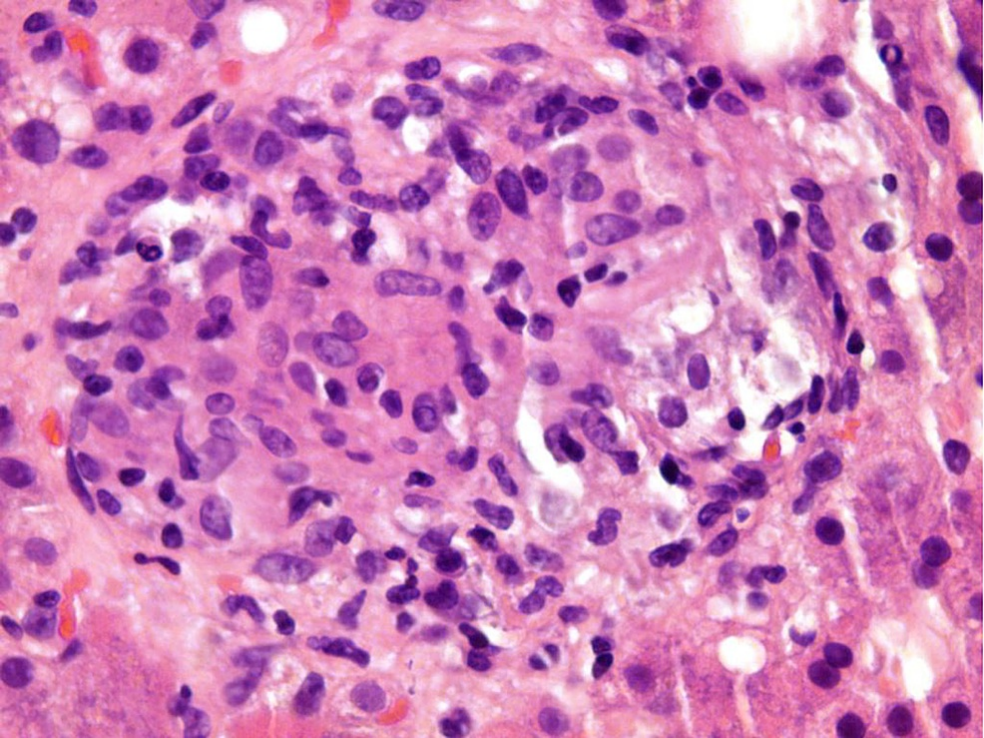
Hematoxylin and eosin stained samples from the patient’s liver biopsy showing non-necrotizing granulomas.

A 57-year-old Mexican female initially presented to the clinic with complaints of urinary incontinence, night sweats, and fevers. Physical examination was unremarkable. Further workup led to a transvaginal ultrasound that showed an unspecified bladder mass. Routine blood work, including complete blood count, basic metabolic panel, and hepatic function panel, were within normal limits. Bladder biopsy was performed, and MB was cultured from the tissue sample. QuantiFERON-TB, serial sputum acid-fast bacilli, and human immunodeficiency virus (HIV) testing were negative. The patient was inappropriately started on RIPE (rifampin, isoniazid, pyrazinamide, ethambutol) therapy and instructed to follow up at the clinic. She was readmitted less than a month later with new symptoms of decreased appetite, weight loss, and dysuria with signs of cholestatic jaundice on laboratory studies (Table 1). Computed tomography (CT) scan of the abdomen showed hepatosplenomegaly (Figure 1). The patient's RIPE therapy regimen was discontinued due to MB resistance to pyrazinamide and due to concerns of drug-induced liver injury. However, the patient’s symptoms failed to improve after discontinuation of the drug. CT- guided biopsy of the liver parenchyma was performed. The histological evaluation showed granulomatous hepatitis with non-necrotizing granulomas positive for rare acid-fast bacilli (Figure 2). Due to a recent history of MB infection, the New York State Department of Health recommended a regimen of ethambutol and linezolid. The patient had significant symptomatic improvement and was discharged with instructions for follow-up in the outpatient setting. The patient received this regimen for one year, and her liver enzymes gradually returned to normal limits.

**Table 1:** Laboratory studies showing cholestatic jaundice pattern

Hospital day	Total bilurubin (mg/dL)	Direct bilirubin (mg/dL)	Alkaline phosphatase (U/L)
Admission	9.1	7.3	792
Hospital day 3	21.2	>10	>12,000

## Discussion

Since 1921, MB has been used to formulate the BCG vaccine used for tuberculosis prevention [2] and more recently as treatment of superficial bladder cancer. Although there is a therapeutic indication for the BCG vaccine, disseminated MB infection is a life-threatening complex, albeit very rare. Extrapulmonary manifestations of MB infection despite being rare are more common than pulmonary manifestations. In patients with this infection, especially those who receive BCG vaccine for superficial bladder cancer, granulomatous hepatitis has been a documented sequela [3]. This form of hepatitis in the setting of MB secondary to BCG vaccine is proposed to be either by hematogenous spread or a hypersensitivity-related mechanism. One prior study conducted in animal models also hypothesized that MB may be disseminated via hematogenous or lymphatic pathways [4]. In the absence of other obvious risk factors, we also hypothesized that there was possible dissemination of mycobacterium that occurred via hematogenous or lymphatic spread after biopsy of the bladder granuloma. The patient had two negative HIV screens occurring seven days apart and multiple series of sputum cultures obtained weeks apart that showed no growth of mycobacterial organisms. It was noted that other possible sources of primary infection were absent as imaging of the thoracic cavity and multiple sputum cultures were negative. Concurrent infection with multiple species of mycobacterial infections is possible; however, taking into account the acute onset of symptoms following bladder biopsy, we can hypothesize that her presentation was a sequelae of this diagnostic procedure.

## Conclusions

This case report will hopefully raise awareness of the possible modes of dissemination of mycobacterial infections. Further investigation may be warranted to evaluate these possible modes of transmission and dissemination as this may lead to prompt diagnosis and treatment of infections. It is also important to note that these infections may affect immunocompetent patients without any obvious risk factors including immunocompromised status and BCG therapy.
